# Impact of Vitamin D on Osseointegration in Dental Implants: A Systematic Review of Human Studies

**DOI:** 10.3390/nu16020209

**Published:** 2024-01-09

**Authors:** Berivan Laura Rebeca Buzatu, Roxana Buzatu, Magda Mihaela Luca

**Affiliations:** 1 Doctoral School, Department of Dental Medicine, “Victor Babes” University of Medicine and Pharmacy Timisoara, Eftimie Murgu Square 2, 300041 Timisoara, Romania; berivan.haj-abdo@umft.ro; 2 Department of Dental Aesthetics, Faculty of Dental Medicine, “Victor Babes” University of Medicine and Pharmacy Timisoara, Eftimie Murgu Square 2, 300041 Timisoara, Romania; 3 Department of Pediatric Dentistry, Faculty of Dental Medicine, “Victor Babes” University of Medicine and Pharmacy Timisoara, Eftimie Murgu Square 2, 300041 Timisoara, Romania; luca.magda@umft.ro

**Keywords:** dental implants, vitamin D, dentistry

## Abstract

This systematic review evaluates the impact of Vitamin D levels on dental implant osseointegration, hypothesizing that optimal Vitamin D enhances success rates, and aims to synthesize data on its relationship with clinical outcomes in implantology. A comprehensive search across PubMed, Cochrane Library, and Web of Science databases included seven peer-reviewed articles meeting the criteria for the review. These studies, conducted between 2008 and 2021, included human subjects and explicitly correlated serum Vitamin D levels with dental implant outcomes, following PRISMA guidelines. The selected studies involved 1462 participants and examined 4450 dental implants. Key findings included a varied implant loss rate ranging from 3.9% to 11.4% across the studies. One study reported a 9.8% implant loss rate, yet found no significant association between Vitamin D receptor polymorphism and implant success. Another study indicated successful implantation following Vitamin D3 supplementation, even in severe deficiency cases. The highest implant loss rate (11.1%) was observed in severely Vitamin D-deficient patients, particularly when compounded by risk factors such as smoking and periodontal disease. Additionally, one study noted significantly improved bone density following post-surgical Vitamin D supplementation for up to 12 weeks. The review supports a link between sufficient Vitamin D levels and successful dental implant osseointegration, suggesting Vitamin D deficiency as a potential risk factor for increased failure and advocating for Vitamin D evaluations in pre-surgical planning to potentially enhance implantology outcomes.

## 1. Introduction

Dental implants have become a cornerstone in the field of restorative dentistry, offering a durable and aesthetically pleasing solution for tooth replacement [1,2]. Globally, the demand for dental implants is on the rise, with a market that was valued at approximately USD 10 billion in 2018 [3] and is projected to reach more than USD 13 billion by the end of 2023 [4], growing at a compound annual growth rate of 6% from 2021 to 2026 [5]. This surge is attributed to an increasing prevalence of dental diseases, rising geriatric population, and growing awareness about oral hygiene, with a 14% increase among elderly individuals [5,6]. Despite the high success rate of dental implants, which is reported to be around 95% [7,8,9], failures due to lack of osseointegration remain a significant challenge [10,11]. Osseointegration, the direct structural and functional connection between living bone and the surface of a load-bearing artificial implant, is critical for the long-term success of dental implants [12].

Recent research has indicated that systemic health factors, particularly nutritional status, can significantly influence the process of osseointegration [13]. Among various nutrients, Vitamin D, a fat-soluble vitamin that is essential for bone health, is particularly noteworthy in the context of bone healing and dental implant success. Its role in regulating calcium and phosphate metabolism makes it a key player in bone remodeling and repair, since it promotes the differentiation and activity of osteoblasts, cells responsible for bone formation, and modulates osteoclasts, which resorb bone tissue [14]. Therefore, adequate levels of Vitamin D are not only essential for maintaining bone density, but also for enhancing the healing process in bone tissues affected by dental implant procedures. Research indicates that optimal Vitamin D levels can accelerate bone healing by improving the quality and quantity of bone formation around the implant, thereby promoting better and faster osseointegration [15]. 

Epidemiological data suggest that Vitamin D deficiency is widespread, affecting about 1 billion people worldwide. This deficiency is especially prevalent in older adults, a group that also forms a significant portion of the dental implant recipient population [16]. The relationship between Vitamin D and osseointegration in dental implants, however, is not fully understood. While some studies suggest that adequate levels of Vitamin D can enhance bone healing and implant integration, others show no significant correlation [17].

The hypotheses of this systematic review are that adequate levels of Vitamin D positively influence the osseointegration of dental implants and that Vitamin D supplementation in deficient individuals can improve the success rate of dental implants. The objective of this study is to conduct a systematic review of the literature to investigate the impact of Vitamin D on the osseointegration of dental implants in humans, and identify the rate of osseointegration, implant success rate, bone density around the implant, and any reported complications in the context of Vitamin D levels in patients. This comprehensive analysis will contribute to a better understanding of the role of Vitamin D in dental implantology and may guide clinical practices regarding nutritional assessment and management in patients undergoing dental implant surgery.

## 2. Materials and Methods

### 2.1. Protocol and Registration

This systematic review aims to analyze the influence of Vitamin D on the osseointegration process in dental implants. We conducted a detailed search across several major electronic databases, including PubMed, Cochrane Library, and Web of Science, with the literature scope extending up to December 2023.

Our search strategy was designed to include a wide range of keywords and phrases, ensuring an exhaustive exploration of all facets related to the study’s objective. The keywords and phrases included “Vitamin D”, “Osseointegration”, “Dental Implants”, “Bone Healing”, “Implant Success”, “Implant Failure”, “Vitamin D Receptors in Bone”, “Bone Metabolism and Implants”, “Oral Rehabilitation”, “Nutritional Status and Dental Surgery”, “Vitamin D Deficiency”, and “Implant Osseointegration in Humans”.

The search strategy combined these terms in various configurations for a thorough literature retrieval. Example search strings used were (“Vitamin D” OR “Cholecalciferol”) AND (“Osseointegration” OR “Bone Integration”) AND (“Implant Failure” OR “Implant Success”) AND (“Dental Implants” OR “Oral Implants”) AND (“Bone Healing” OR “Bone Metabolism”) AND (“Human Studies” OR “Clinical Outcomes”). 

This review adheres to the Preferred Reporting Items for Systematic Reviews and Meta-Analyses (PRISMA) guidelines and was registered with the International Prospective Register of Systematic Reviews (PROSPERO) [18]. Our aim is to maintain a structured, transparent, and reproducible methodological approach. Moreover, this review was registered on the Open Science Framework to ensure open access to our methodology and findings, with the registration code osf.io/dx5sm.

### 2.2. Eligibility Criteria and Definitions

For the systematic review, we established specific eligibility criteria to ensure a focused and relevant analysis. Each study was initially screened for duplicates, followed by a detailed assessment of abstracts by two independent researchers to confirm relevance to our research objectives. Any selection discrepancies were resolved through consultation with a third researcher.

The Inclusion criteria were defined as follows: (1) studies must specifically investigate the impact of Vitamin D on osseointegration in dental implants in human subjects; (2) research must include the measurement of serum Vitamin D levels in participants, and correlate these levels with the success rate of dental implants; (3) studies should provide a clear and detailed methodology, including how Vitamin D levels were measured and how osseointegration was assessed; (4) only peer-reviewed articles published in English with explicit details on outcomes related to the success of dental implants and Vitamin D levels should be included.

The exclusion criteria encompassed (1) non-human studies, i.e., a ny research that does not involve human participants or focuses on in vitro or animal models; (2) lack of specific focus, i.e., studies not specifically examining the relationship between Vitamin D levels and osseointegration in dental implants, or only studying these separately; (3) insufficient data on vitamin D and osseointegration, i.e., esearch that does not provide clear outcome measures related to Vitamin D levels and the success of dental implants; and (4) non-peer-reviewed sources, which meant the exclusion of non-peer-reviewed articles, including preprints, conference proceedings, general reviews, commentaries, and editorials.

According to guidelines and consensus from professional dental associations and research studies [19], osseointegration is characterized by (1) biocompatibility: when the implant material does not cause a rejection response from the body’s immune system; (2) the bone–implant interface: osseointegration requires direct bone-to-implant contact without interposing soft tissue at the microscopic level, critical for the stability and longevity of the implant; (3) stability and lack of mobility: the implant must achieve primary stability through mechanical means (i.e., the surgical technique and immediate fitting of the implant in the bone) and secondary stability through biological means (bone growth and remodeling around the implant); (4) the timeframe for osseointegration: the process of osseointegration typically takes several weeks to months, during which the bone cells attach directly to the implant surface, effectively locking the implant into the jawbone; (5) the assessment of osseointegration, which occurs through clinical assessments (such as implant stability and absence of pain or infection), radiographic analysis (to observe bone–implant contact and bone quality), or histological examinations to confirm direct bone contact at the microscopic level; and (6) success criteria, which include the absence of peri-implant radiolucency, no persistent pain or discomfort or infection, and the functional loading of the implant without any signs of mobility.

### 2.3. Data Collection Process

In this systematic review, the initial database search yielded 677 articles. Of the total number of screened articles (195), 106 were identified as duplicates and removed. The remaining articles underwent a preliminary screening based on abstracts, leading to the exclusion of non-relevant studies. This was followed by a thorough full-text review of the shortlisted articles by two authors, with disagreements resolved by a third author to ensure accuracy and objectivity.

**Figure 1:**
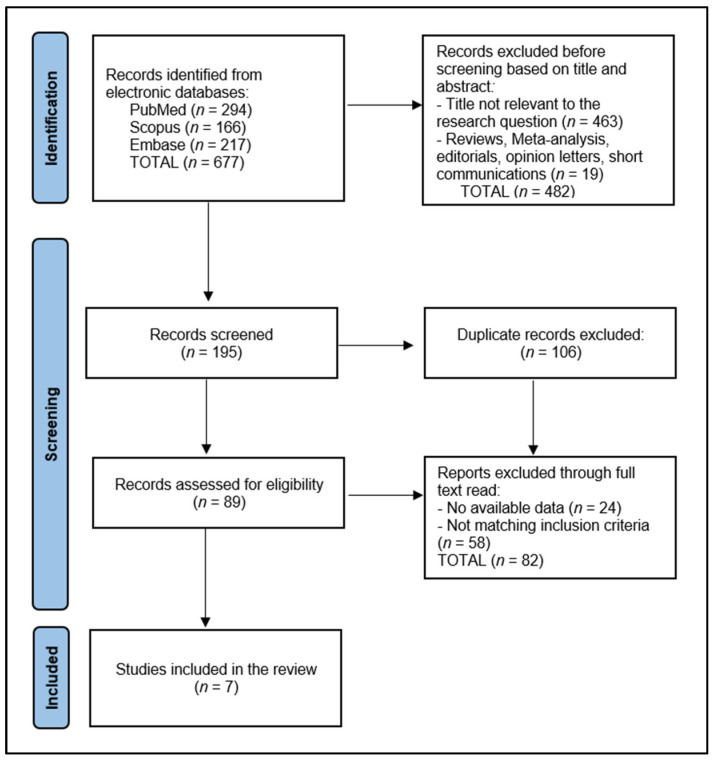
PRISMA flow diagram.

A total of seven articles met our stringent criteria and were included in the review. The data extraction process, conducted by two researchers (R.B. and B.A.B.), involved gathering information on study design, participant demographics, implant type, osseointegration features, vitamin D assessment, and outcomes related to vitamin D levels in relation to osseointegration of the dental implant, as presented in Figure 1.

### 2.4. Quality Assessment

For quality assessment, we employed the Newcastle–Ottawa Scale for cohort studies and the Cochrane Collaboration’s tool for randomized trials [20]. Each study was independently evaluated by two researchers, with scores indicating the quality of the studies: low, medium, or high. This approach ensured an unbiased evaluation of the selected literature, forming the basis for our systematic analysis.

## 3. Results

### 3.1. Study Characteristics

The systematic review explored the impact of Vitamin D on osseointegration in dental implants, as detailed in Table 1, encompassing seven studies. These studies, conducted in a diverse range of countries including Brazil, the USA, the United Kingdom, Germany, Italy, and Poland, indicate a broad international focus on the subject. The research spanned from 2008 to 2021, highlighting an ongoing interest in the field over more than a decade. The earliest study in this review, conducted by Alvim-Pereira et al. in Brazil, was published in 2008, while the most recent study by Kwiatek et al. from Poland appeared in 2021.

The studies varied in design, ranging from case reports and case–control studies to a randomized trial. Specifically, two studies were case–control [21,26], three were case reports [22,23,24], one was a case series [24], one a retrospective cohort study [25], and one a randomized trial [27]. This assortment of study designs reflects the multifaceted approach researchers have taken to examine the relationship between Vitamin D and dental implant osseointegration. In terms of quality of evidence, the review found a mix. Two studies [21,26] from Brazil and one from Italy [25] were rated as having a medium quality of evidence. In contrast, three studies [22,23,24]—from the USA, the United Kingdom, and Germany—were categorized as low in evidence quality. Notably, the study from Poland [27] stood out as the only one classified with high-quality evidence, which was also the sole randomized trial in the review. 

### 3.2. Participants’ Characteristics

Table 2 provides a comprehensive overview of the characteristics of study participants across seven studies. The total number of individuals involved in these studies was 1462, highlighting a diverse range of participant cohorts. The largest study, conducted by Mangano et al. [25], included 885 individuals, whereas three studies by Flanagan et al. [22], Bryce et al. [23], and Fretwurst et al. [24] were case studies with 1, 1, and 2 participants, respectively. The participant groups varied significantly across the studies. Alvim-Pereira et al. [21] compared 137 individuals with dental implants to 70 without, while Pereira et al. [26] studied 81 individuals with a Vitamin D receptor mutation against 163 controls. Kwiatek et al. [27] categorized their 122 participants into three groups based on Vitamin D levels and supplementation.

Participants’ average age ranged from 29 years in Bryce et al.’s study [23] to 57.3 years in Mangano et al.’s research [25]. Regarding gender distribution, the studies predominantly featured male participants. In the study of Alvim-Pereira et al. [21], 36.5% of participants with implants and 40.0% without implants were male. In contrast, Mangano et al. [25] reported a slightly higher proportion of male participants, at 51.5%. The case studies by Flanagan et al. [22], Bryce et al. [23], and Fretwurst et al. [24] each involved male participants exclusively.

### 3.3. Implant Assessment

The total number of implants considered in these studies was significant, having a total of 4450 studied dental implants, with Alvim-Pereira et al. [21] alone accounting for 1367 implants, of which 1232 were healthy and 135 were lost. The study by Mangano et al. [25] involved 1740 implants, and that of Pereira et al. [26] included 1193 implants. Smaller-scale studies, such as Bryce et al. [23] and Flanagan et al. [22], examined 1 and 18 implants, respectively.

Location-wise, there was a notable distinction in implant placement between healthy and lost implants in Alvim-Pereira et al.’s study [21], with 50.9% of healthy implants in the maxilla and 55.4% in the posterior region, compared to 37.5% and 64.7%, respectively, for lost implants. Kwiatek et al. [27] reported specific locations within the mandible, with premolars accounting for 25.8%, and molars for 74.2%. The size of the implants varied across studies. For instance, Alvim-Pereira et al. [21] reported an average diameter of 3.99 mm and length of 13.16 mm for healthy implants, compared to a diameter of 4.03 mm and length of 12.35 mm for lost implants. In contrast, Bryce et al. [23] used a titanium implant with a diameter of 4.3 mm and length of 10 mm.

Follow-up duration was another critical aspect, with studies ranging from 4 months (Mangano et al. [25]) to an estimated survival of 250 weeks (Pereira et al. [26]). Alvim-Pereira et al. [21] had an average follow-up of 43 weeks, while Flanagan et al. [22] and Kwiatek et al. [27] observed their implants for 7 and 6–12 weeks, respectively (Table 3).

### 3.4. Outcomes

The findings from these studies demonstrated diverse effects of Vitamin D on dental implant survival and provided a nuanced understanding of risk factors associated with osseointegration. In the study by Alvim-Pereira et al. [21], a 9.8% implant loss rate was observed, but no significant association was found between Vitamin D TaqI receptor polymorphism and implant loss, suggesting that genetic variations in the Vitamin D receptor may not be a critical factor in the success of dental implants. Flanagan et al. [22] demonstrated successful implantation after extraction in a patient undergoing Vitamin D3 supplementation and other treatments, indicating that low serum Vitamin D levels might not pose a significant risk for implant loss if other factors, like calcium levels, are adequately managed.

Bryce et al. [23] showed successful implantation in the context of severe Vitamin D deficiency, implying that low Vitamin D might contribute to challenges in osseointegration, but isn’t an insurmountable barrier. Fretwurst et al. [24] observed a failure in two out of nine implants in the context of Vitamin D deficiency, but successful replacement was achieved following Vitamin D supplementation, highlighting the potential benefit of addressing Vitamin D levels for implant success.

**Figure 2:**
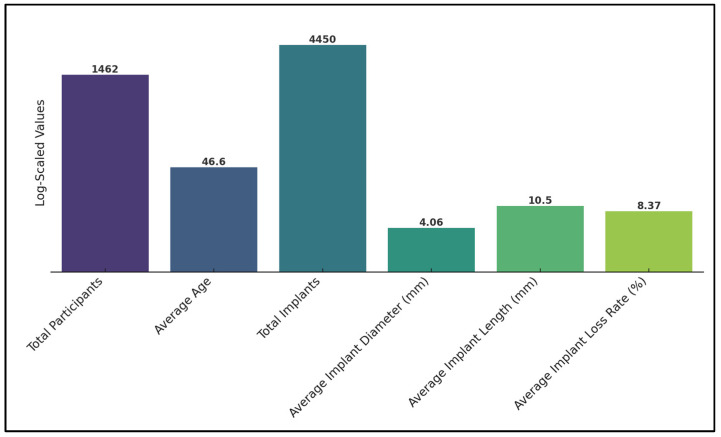
Summary of findings (logarithmically scaled).

Mangano et al. [25] reported a 3.9% overall implant loss rate, which increased to 11.1% among patients with severe vitamin D deficiency. This study indicated a four times higher prevalence of early implant loss with low serum Vitamin D levels, especially when associated with smoking and periodontal disease. Pereira et al. [26] found an 11.4% implant loss rate in the context of Vitamin D deficiency due to a receptor mutation. The study specifically identified the Vitamin D allele G of rs3782905 as being significantly associated with poor osseointegration, suggesting a genetic component to Vitamin D’s impact on implant success. Finally, Kwiatek et al. [27] did not report specific implant loss rates, but noted significantly higher bone density in patients with Vitamin D supplementation after 12 weeks, implying a beneficial effect of Vitamin D on bone quality (Table 4). Overall, the average implant loss rate across all studies was 8.37%, as presented in Figure 2.

## 4. Discussion

### 4.1. Summary of Evidence

The systematic review on the impact of Vitamin D on osseointegration in dental implants provides a compelling insight into the nuanced relationship between Vitamin D and dental implant success. The diverse study designs and participant demographics across the seven studies reviewed highlight a global research interest in this area, underscoring the significance of Vitamin D in dental implantology. One of the most interesting findings comes from Alvim-Pereira et al. [21], which shows a 9.8% implant loss rate but finds no significant association between Vitamin D TaqI receptor polymorphism and implant loss. This suggests that while Vitamin D plays a role in osseointegration, its impact might not be directly tied to genetic variations in the Vitamin D receptor, indicating a more complex interaction between Vitamin D and bone remodeling than previously thought [28,29]. On the other hand, studies such as those by Flanagan et al. [22] and Fretwurst et al. [24] demonstrate successful implantation outcomes in the context of Vitamin D supplementation, indicating that managing Vitamin D levels can positively influence implant success.

The study by Mangano et al. [25] is particularly noteworthy, highlighting a four-fold increase in early implant loss in patients with low serum Vitamin D levels. This correlation, particularly when considered alongside the additional risk factors of smoking and periodontal disease, underlines the importance of a holistic approach to patient health in dental implantology, as additional factors such as smoking can significantly increase implant failure rate by more than 140% [30]. Similarly, the findings from Pereira et al. [26], showing a significant association between a specific Vitamin D allele and poor osseointegration, suggest a potential genetic predisposition that could influence patient-specific treatment strategies. Kwiatek et al. [27], in their high-quality randomized trial, present another dimension to this discussion by noting a significant increase in bone density following Vitamin D supplementation. This finding provides a tangible link between Vitamin D levels and bone health, reinforcing the idea that Vitamin D supplementation could be a key factor in improving osseointegration outcomes.

The review revealed diversity in implant characteristics, including variations in the number of implants studied, their locations, sizes, and follow-up periods. This diversity could reflect the wide range of clinical practices and patient populations involved in these studies. The differences in implant success rates between the maxilla and posterior region, as observed in Alvim-Pereira et al.’s study [21], may suggest anatomical particularities in osseointegration, as one study showed a significantly higher rate of implant osseointegration in the anterior maxilla, from approximately 90% to 70% in the posterior maxilla [31]. Furthermore, the varying follow-up durations indicate differing methodologies in assessing long-term implant success, emphasizing the need for standardized follow-up periods in future research.

The analyzed studies reviewed varied approaches to Vitamin D supplementation, with few providing explicit details on the regimen. Notably, Kwiatek et al. [27] monitored Vitamin D levels from 23.9 ng/mL on the day of surgery to 33.1 ng/mL after 12 weeks, indicating ongoing supplementation post-surgery. Flanagan et al. [22] used Vitamin D3 supplements alongside other treatments. Fretwurst et al. [24] did not mention the exact regimen of the supplementation, but it is noted that after supplementation, one patient’s vitamin D level increased to 46 μg/L, indicating a significant improvement. The time frame between the supplementation and the subsequent successful implant surgery is mentioned for one patient as 6 months, suggesting a period for the vitamin D levels to increase and possibly stabilize before the next implant attempt. However, specific details on the duration before surgery and target levels were generally not provided, reflecting a diversity in practices and a lack of standardized protocols across the studies. Although the exact supplementation protocols varied across studies, and no single approach was uniformly validated, these findings suggest that careful management of Vitamin D levels could be a critical factor in improving osseointegration and implant success. Compared to human research, in vitro and animal studies on the impact of Vitamin D on dental implant osseointegration are more common, and show more diverse outcomes. Plenty of animal studies have demonstrated that systemic Vitamin D supplementation enhanced new bone formation around implants; however, this finding was not universal [32,33]. Another study with healthy animals supplemented with Vitamin D reported only a minor improvement in osseointegration [34]. Furthermore, it was noted that patients who are either sufficient or insufficient in Vitamin D derived less benefit from supplementation compared to those who are deficient [35], with high-dose Vitamin D supplementation potentially reducing bone mineral density in healthy subjects [36].

Several chronic diseases such as diabetes mellitus predispose individuals to lower dental implant success and shorter life duration. However, in diabetic animal models, some studies have examined the effect of Vitamin D supplementation on osseointegration, although no significant difference was observed in bone-to-implant contact and new bone formation between Vitamin D-supplemented and non-supplemented diabetic rats [37]. However, when insulin treatment was combined with Vitamin D supplementation, one study reported a significant increase in bone area and osseointegration [32]. This indicates a potential benefit of combined therapy in diabetic conditions. Additionally, Vitamin D supplementation periods in animal studies varied, with some starting 7 days post surgery and others up to 8 weeks pre-surgery and 4 weeks post surgery [38]. One study on animals found that the group deficient in Vitamin D had significantly lower cortical BIC compared to the group supplemented for 8 weeks pre-surgery and 14 days post surgery [39]. Another group supplemented only 14 days pre-surgery and post surgery showed no significant change in BIC, but had a notable decrease in bone volume/total volume. Therefore, these varied findings reflect the lack of a standardized approach to Vitamin D supplementation in both animal and human research [40,41].

### 4.2. Limitations

Our systematic review, aiming to elucidate the impact of Vitamin D on the osseointegration of dental implants, presents several limitations that must be acknowledged. Firstly, there was notable heterogeneity among the studies included, especially in terms of study designs, methods of Vitamin D assessment, and evaluation of osseointegration. This diversity, spanning case reports to randomized trials and including various participant demographics and methodologies, could impact the comparability and consistency of the findings across studies. Secondly, the potential for publication bias exists, given the relatively small number of studies that met our stringent inclusion criteria. Additionally, the restriction to English-language, peer-reviewed articles might have introduced a language bias, possibly excluding relevant research published in other languages or non-peer-reviewed formats.

Another limitation concerns the variation in data reporting across the included studies. Some studies lacked comprehensive data on Vitamin D levels or detailed information on the osseointegration process, which might constrain the depth of our analysis and the strength of the conclusions drawn. Moreover, the scope of our review was limited to human studies, excluding potentially valuable insights from animal or in vitro models. Finally, the observational nature of most included studies limits the ability to infer causality between Vitamin D levels and dental implant success. To establish a more concrete relationship, future research should focus on prospective and intervention studies that can provide stronger evidence for causal links and help refine clinical guidelines for Vitamin D supplementation in patients undergoing dental implant surgery.

## 5. Conclusions

This systematic review reveals no significant association between Vitamin D levels and the osseointegration of dental implants, highlighting a notable heterogeneity among the included studies. This variability underlines the complexity of establishing a direct correlation in human studies, contrasting with the more prevalent and consistent findings in animal research. The results underscore the necessity for a more standardized approach in evaluating the impact of Vitamin D on dental implant success. Current evidence suggests that while Vitamin D status may have a role in bone health, and its association with several risk factors can impact the osseointegration and cause implant failure, its direct influence on dental implant osseointegration remains inconclusive. These findings advocate for further research with more uniform methodologies to clarify the relationship between Vitamin D and osseointegration in dental implantology.

## Figures and Tables

**Table 1:** Study characteristics.

Study and Author	Country	Study Year	Study Design	Quality of Evidence
Alvim-Pereira et al. [21]	Brazil	2008	Case-Control	Medium
Flanagan et al. [22]	USA	2013	Case Report	Low
Bryce et al. [23]	United Kingdom	2014	Case Report	Low
Fretwurst et al. [24]	Germany	2016	Case Series	Low
Mangano et al. [25]	Italy	2018	Retrospective Cohort	Medium
Pereira et al. [26]	Brazil	2019	Case-Control	Medium
Kwiatek et al. [27]	Poland	2021	Randomized Trial	High

**Table 2:** Characteristics of study participants.

Study Number	Number of Individuals	Study Groups	Age (Years)	Sex (Male)
Alvim-Pereira et al. [21]	207	137 with dental implants vs. 70 without implants	51.6 years vs. 52.8 years	36.5% with implants vs. 40.0% without
Flanagan et al. [22]	1	NR	34 years	1 Male
Bryce et al. [23]	1	NR	29 years	1 Male
Fretwurst et al. [24]	2	NR	48 years vs. 51 years	2 Male
Mangano et al. [25]	885	850 successful implants vs. 35 early failures	57.3 years	51.5%
Pereira et al. [26]	244	81 with Vitamin D receptor mutation vs. 163 controls	51.9 years	33.6%
Kwiatek et al. [27]	122	Group 1: Vitamin D < 30 ng/mL, Group 2: Vitamin D < 30 ng/mL + supplement, Group 3: Vitamin D > 30 ng/mL	43.8 years	46.7%

NR—not reported.

**Table 3:** Characteristics of implants.

Study Number	Number of Implants	Location (Mandible, Maxilla)	Implant Size (Diameter, Length)	Follow-Up
Alvim-Pereira et al. [21]	1232 healthy implants, 135 lost implants	Healthy implants: 50.9% maxilla, 55.4% posterior region Lost implants: 37.5% maxilla, 64.7% posterior region	Healthy implants: diameter 3.99, length 13.16 Lost implants: diameter: 4.03, length 12.35	43 weeks
Flanagan et al. [22]	18	Mandible and maxilla	NR	7 months
Bryce et al. [23]	1	Mandibular premolar	Diameter: 4.3, length 10	5 months
Fretwurst et al. [24]	9	Mandible	Diameter: 4.1, length 12 vs. Diameter 4.3, length 7	NR
Mangano et al. [25]	1740	Mandible and maxilla	NR	4 months
Pereira et al. [26]	1193	NR	NR	250 weeks
Kwiatek et al. [27]	122	Mandible: premolars 25.8%, molars 74.2%	Diameter: 3.3–4.2, length: 8–11.5	6–12 weeks

NR—not reported; vitamin D hypovitaminosis (insufficiency) is considered below 20 ng/mL or 50 nmol/L; vitamin D deficiency is considered below 10 ng/mL or 25–30 nmol/L.

**Table 4:** Study outcomes.

Study Number	Vitamin D Assessment	Outcomes	Risk Factors
Alvim-Pereira et al. [21]	NR	9.8% implant loss rate	No association between Vitamin D TaqI receptor polymorphism and implant loss.
Flanagan et al. [22]	Vitamin D3 supplementation, phosphate binders and calcium cinacalcet calcimimetic, 3 × dialysis/week	Successful implantation	Low serum Vitamin D levels do not pose an elevated risk for loss of dental implant if calcium levels are properly corrected in patients with IgA nephropathy.
Bryce et al. [23]	Severe Vitamin D deficiency	Successful implantation	Low serum Vitamin D might contribute to unsuccessful osseointegration in dental implants.
Fretwurst et al. [24]	Vitamin D deficiency	22.2% implant loss rate	Successful replacement after Vitamin D supplementation
Mangano et al. [25]	Vitamin D deficiency in 53.7% of patients; 29.5 ng/mL vs. 25.4 ng/mL in the early failure group	3.9% implant loss rate; 11.1% among patients with severe vitamin D deficiency	Four times higher prevalence of early implant loss with low serum Vitamin D levels; higher when associated with smoking and periodontal disease.
Pereira et al. [26]	Vitamin D deficiency due to receptor mutation	11.4% implant loss rate	Vitamin D allele G of rs3782905 significantly associated with poor osseointegration.
Kwiatek et al. [27]	Day of surgery: 23.9 ng/mL, After 6 weeks: 30.4 ng/mL, After 12 weeks: 33.1 ng/mL	NR	Significantly higher bone density in patients with Vitamin D supplementation after 12 weeks.

NR—not reported; OR—odds ratio. Vitamin D hypovitaminosis (insufficiency) is considered below 20 ng/mL or 50 nmol/L; vitamin D deficiency is considered below 10 ng/mL or 25–30 nmol/L; severe deficiency is considered for levels below 10 nmol/L.

## Data Availability

Data is contained within the article.
